# A Multienzyme Magnetic Nanocatalyst for Efficient, Sustainable Hydrolysis of Wastewater-Grown Microalgae Consortia

**DOI:** 10.1021/acssuschemeng.5c08509

**Published:** 2025-12-17

**Authors:** Suvidha Gupta, Jorge M. Marchetti

**Affiliations:** Faculty of Science and Technology, Realtek, Norwegian University of Life Sciences, Drøbakveien 31, 1432 Ås, Norway

**Keywords:** enzyme immobilization, response surface methodology, saccharification, enzyme reusability, circular bioeconomy

## Abstract

The rigid cell walls of microalgal consortia (*Scenedesmus* and *Tribonema*) hinder the efficient release of intracellular bioproducts and present a significant challenge for sustainable biomass utilization. To address this challenge, a novel multienzyme magnetic nanocatalyst (ME-MNC) was synthesized to enhance enzymatic hydrolysis. It comprised cellulase, α-amylase, amyloglucosidase, and alcalase immobilized on amino-functionalized iron oxide nanoparticles. Optimization using Central Composite Design identified optimal parameters: 103.82 mM glutaraldehyde, 178.79 min cross-linking time, and 7.08 h immobilization time. The catalytic system demonstrated enzyme activity recoveries of 64.66% (cellulase), 67.02% (α-amylase), 43.40% (amyloglucosidase), and 81.14% (alcalase). Structural and physicochemical characterizations, including X-ray diffraction, Fourier transform infrared spectroscopy, field-emission scanning electron microscopy, and thermogravimetric analysis, confirmed successful synthesis and enzyme immobilization. Under optimal conditions, the ME-MNC achieved 81.7% sugar recovery, exceeding the yield of the free enzyme mixtures (69.5%), and maintained stable performance across pH 6–8 and temperatures of 50–60 °C. After five reuse cycles, the catalyst retained 81% sugar recovery and 96% protein recovery, underscoring its efficiency and reusability. This reusable catalyst offers a sustainable and scalable strategy for microalgal biomass conversion, enabling efficient sugar recovery for biobased chemical production, with additional potential applications in biofuel production and other microalgae-based industries.

## Graphical abstract

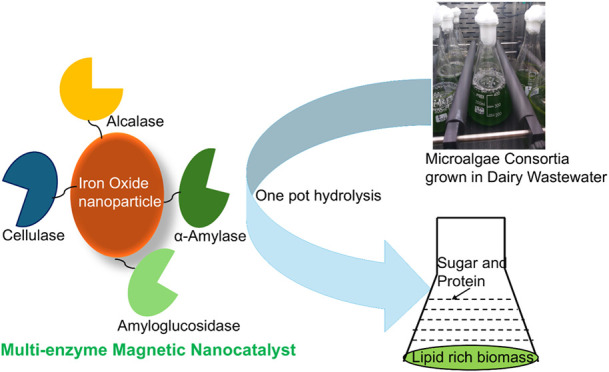

## Introduction

Microalgae are emerging as promising feedstocks for biofuel and biochemical production. This is due to the abundance of high-value components, such as lipids, proteins, and carbohydrates, in microalgae. Wastewater-grown microalgae, in particular, offer dual benefits of bioremediation and bioresource recovery.^1,2^ Among different waste streams, dairy wastewater (DW) holds particular promise as a growth medium because of its high organic load and nutrient content (nitrogen and phosphorus). Cultivating microalgae in DW not only treats the effluent by reducing both organic and inorganic contents but also produces nutrient-rich biomass suitable for further valorization. This symbiotic approach addresses both environmental pollution and sustainable biomass generation, providing a cost-effective and eco-friendly solution.^1,2^ Despite these advantages, the efficient hydrolysis of microalgal biomass remains a major challenge. The rigid and heterogeneous composition of the microalgal cell wall makes it difficult to release intracellular components. As a result, hydrolysis is often the most energy- and cost-intensive step in biorefinery operations.^3,4^ Enzymatic hydrolysis has been proposed as a greener and more precise alternative to traditional chemical and physical methods. The chemical and physical methods are often energy-consuming and generate undesirable byproducts. Efficient hydrolysis typically requires a combination of enzymes, such as cellulase, amylase, amyloglucosidase, and protease. These enzymes are used to degrade the various components of the microalgal cell wall and release intracellular components.^5–7^ Despite its green credentials, conventional enzymatic hydrolysis has several limitations. These include multistep processing, lengthy reaction times, high operational complexity, enzyme instability, and poor reusability.^4,5^ Overcoming these challenges is essential to unlocking the full potential of wastewater-grown microalgae. One promising strategy is enzyme immobilization on suitable supports, which can enhance enzyme stability, activity, and reusability, thereby making hydrolysis more efficient and cost-effective.^8–12^

Iron oxide nanoparticles (IONPs) have emerged as ideal support for enzyme immobilization. Their large surface area facilitates a high enzyme loading. Their magnetic properties enable easy recovery and reuse. Their biocompatibility supports the creation of multienzyme systems.^9,10^ Prior studies have demonstrated the successful immobilization of individual or dual enzymes for microalgal biomass hydrolysis. For example, cellulase immobilized on MgO–Fe_3_O_4_ nanoparticles enhanced cellulose hydrolysis,^8^ while coimmobilized cellulase and lysozyme on IONPs improved lipid extraction.^10^ These studies also reported improved pH and thermal stability of immobilized enzymes compared with free enzymes.^8,10^ While IONPs have been widely utilized as supports for enzyme immobilization, most research has focused on relatively simple substrates or single-species microalgal systems. Applications involving enzyme immobilization on IONPs for the hydrolysis of complex biomass, such as microalgal consortia cultivated in real wastewater, remain comparatively limited. Moreover, existing studies typically employ single or dual enzyme systems that target either sugar or lipid recovery. Integrating multiple enzymes with complementary catalytic functions into a single, magnetically recoverable nanocatalyst could significantly enhance the hydrolysis efficiency and streamline biomass conversion. To date, no studies have explored a multienzyme magnetic nanocatalyst (ME-MNC) strategy for the one-pot hydrolysis of microalgal consortia grown in DW. Such a system could simultaneously release fermentable sugars and proteins, while preserving lipid-rich biomass for downstream valorization. This concept underpins the development of the ME-MNC in the present study.

To address this gap, the present study introduces a novel ME-MNC that coimmobilizes cellulase, amylase, amyloglucosidase, and alcalase onto IONPs. This innovative system enables simultaneous hydrolysis of diverse components in microalgal biomass, consolidating traditionally sequential processes into a single step. This approach not only enhances the reaction efficiency but also reduces operational complexity, waste generation, and energy consumption. It thereby offers a more sustainable and cost-effective solution for microalgal bioprocessing.^9^

To achieve maximum enzyme activity recovery (EAR), key parameters such as glutaraldehyde concentration (GC), cross-linking time (CT), and immobilization time (IT) were optimized by using response surface methodology (RSM). The developed ME-MNC was then characterized using Fourier transform infrared spectroscopy (FTIR), X-ray diffraction (XRD), field-emission scanning electron microscopy (FE-SEM), and thermogravimetric analysis (TGA). Each method was chosen to provide detailed information about functional groups, crystallinity, morphology, and thermal properties. Subsequently, the ME-MNC’s efficacy was evaluated for hydrolyzing microalgal consortia grown in DW. Hydrolysis conditions, including pH and temperature, were optimized to maximize the release of reducing sugars and proteins. Finally, the reusability of the ME-MNC was assessed over five hydrolysis cycles under optimized conditions, demonstrating its performance stability and sustainability. This study provides a scalable and innovative platform to efficiently process wastewater-grown microalgal biomass to produce valuable resources. The findings pave the way for continued advances in sustainable biofuel and biochemical production.

## Materials and Methods

### Materials

IONPs were purchased from Nanographi Pvt. Ltd. Commercial enzymes cellulase, from *Trichoderma reesei* (C2730), α-amylase from *Bacillus* sp. (A4862), amyloglucosidase from *Aspergillus niger* (A7095), and alcalase (Alcalase Enzyme, *Bacillus licheniformis*), were purchased from Sigma-Aldrich. All other reagents were purchased from VWR, Norway.

### Preparation of the Multienzyme Magnetic Nanocatalyst

The ME-MNC was prepared by coimmobilizing cellulase, α-amylase, amyloglucosidase, and alcalase onto amino-functionalized, glutaraldehyde-activated IONPs. Preparation parameters, including GC, CT, and IT, were optimized to maximize EAR. The activity recovery of each enzyme was determined using standard activity assays, while immobilization efficiency was assessed by measuring protein content in wash supernatants using the Bradford assay.^13^ Detailed synthesis and experimental procedures are described in the Supporting Information.

### Characterization of the Multienzyme Magnetic Nanocatalyst

Structural and physicochemical properties of the ME-MNC were characterized using FTIR, XRD, FE-SEM, and TGA, with detailed protocols provided in the Supporting Information.

### Design of Experiments and Statistical Analysis

To achieve maximum activity recovery for each enzyme, GC, CT, and IT were optimized using a Central Composite Design (CCD), as presented in Table 1. A total of 20 experimental runs were conducted, comprising 6 central points, 8 linear points, and 6 nonlinear points. Statistical analysis was performed using Statgraphics Centurion XVII (version 17.2.04).

**Table 1:** Levels of the Independent Variables Used in the CCD

		levels				
independent variables	symbols	**–**α	**–**1	0	1	α
glutaraldehyde concentration (mM)	A	35.68	80	145	210	254.32
cross-linking time (min)	B	79.09	120	180	240	280.91
immobilization time (h)	C	3.95	6	9	12	14.05

### Determination of Enzyme Activity

Enzymatic activities were determined using 1% (w/v) substrate solutions of carboxymethyl cellulose (CMC) for cellulase and soluble starch for both α-amylase and amyloglucosidase. All substrate solutions were prepared in 50 mM acetate buffer containing 10 mM CaCl_2_ at pH 5.^14–18^ In all cases, the reactions were terminated by adding dinitrosalicylic acid (DNS) reagent. Reducing sugar concentrations were quantified using the DNS method by measuring the absorbance at 540 nm. Results were expressed as glucose equivalents for cellulase and amyloglucosidase, and as maltose equivalents for α-amylase.^15,18,19^ For the determination of alcalase enzyme activity, 0.65% (w/v) casein was used as the substrate, following Sigma-Aldrich’s protocol.^20^ All experimental analyses were carried out in duplicate.

### Preparation of Microalgal Biomass for Enzymatic Hydrolysis

A consortium of microalgae, comprising *Scenedesmus* and *Tribonema* in a 1:1 ratio, was cultivated in DW, characterized by a Chemical Oxygen Demand of 952 ± 15 mg L^–1^, NO_3_^–^–N of 8.9 ± 0.8 mg L^–1^, and PO_4_^3–^–P of 3.4 ± 0.007 mg L^–1^. The cultivation period lasted 15 days, yielding a biomass concentration of 520 ± 7 mg L^–1^.^1^ After cultivation, the biomass was harvested through sedimentation, washed three times with distilled water, and freeze-dried to prepare it for enzymatic hydrolysis. The biomass was analyzed for molecular composition, revealing carbohydrate content of 53.3 ± 1.8%, lipid content of 13.2 ± 0.1%, and protein content of 12.3 ± 0.5%.^1^

### Application of ME-MNC for Hydrolysis of Wastewater-Grown Microalgal Biomass

To evaluate the efficacy of a multienzyme magnetic nanocatalyst for the hydrolysis of microalgal biomass, the biomass was prepared as a 10% w/v suspension in 0.5 M HCl. The prepared suspension was autoclaved at 121 °C for 30 min. After autoclaving, the pH of the hydrolyzed biomass mixture was adjusted to target values ranging from 4 to 10 by adding 2 M NaOH. To avoid volume changes from pH adjustment, the solvent was subsequently evaporated under a gentle nitrogen stream at 50 °C, concentrating the mixture for further processing. Once evaporation was complete, the respective pH buffers were added to each experimental tube to maintain a 10% (w/v) suspension: acetate buffer for pH 4 and 5, phosphate buffer for pH 6–8, and glycine-NaOH buffer for pH 9 and 10. To assess the effects of ME-MNC on cell wall disruption and recovery of sugars and proteins, a control experiment using a free enzyme mixture (FEM) was also conducted. This control comprised a mixture of cellulase, amylase, amyloglucosidase, and alcalase in a 1:1:1:1 ratio with equal protein concentration.

For temperature optimization, the pH was fixed at 8 for ME-MNC and 5 for FEM based on the pH optimization results. Enzymatic hydrolysis was then carried out at temperatures ranging from 40 to 70 °C. In all experiments, 1% (w/v) ME-MNC or 1% (v/v) FEM was added to the microalgae suspension, and the reaction mixtures were incubated in a shaking water bath at 150 rpm for 24 h at the target temperature (50 °C for pH studies). After 24 h, the samples were filtered through a 0.45 μm syringe filter to remove residual solids or particulates. Filtrates were collected, and hydrolysis efficiency was determined by analyzing reducing sugar content using the DNS method and protein recovery using the Bradford method.^13,19^ All experimental analyses were carried out in duplicate. Equation 1 was used to determine the recovery (%) of sugar and protein:

1 $$\mathrm{recovery}\;\left(\%\right)=\frac{\mathrm{concentration}\;\mathrm{of}\;\mathrm{reducing}\;\mathrm{sugar}\;\mathrm{or}\;\mathrm{protein}\;\mathrm{in}\;\mathrm{filtrate}}{\mathrm{concentration}\;\mathrm{of}\;\mathrm{total}\;\mathrm{carbohydrate}\;\mathrm{or}\;\mathrm{protein}\;\mathrm{in}\;\mathrm{microalgal}\;\mathrm{biomass}}\times 100$$

### Recycling and Reusability of the Multienzyme Magnetic Nanocatalyst

To evaluate the reusability of the ME-MNC, the catalyst was separated from the biomass mixture at the end of each 24 h hydrolysis cycle using a magnet. After separation, the ME-MNC was washed three times with buffer to remove residual biomass or reaction byproducts. The washed nanocatalyst was then resuspended in a fresh biomass mixture under optimized conditions (10% biomass concentration, pH 8, and 50 °C) and incubated for another 24 h in a shaking water bath at 150 rpm. This process was repeated for five cycles. At the end of each cycle, samples were collected, filtered through a 0.45 μm syringe filter, and analyzed for reducing sugars and protein recovery to assess any loss in catalytic activity and recovery performance across multiple uses. The sugar and protein recovery in the first cycle was considered as 100%, and relative recovery percentages were evaluated for subsequent cycles. All experimental analyses were carried out in duplicate.

## Results and Discussion

### Design of Experiments

#### Effect of Variables

The effect of GC, CT, and IT on the EAR is shown in Figure 1. The results indicate that these independent variables significantly influenced the activity recovery of cellulase, α-amylase, amyloglucosidase, and alcalase. Cellulase and α-amylase showed the highest activity recovery at 145 mM GC, 180 min of CT, and 9 h of IT, with a decline in activity recovery observed beyond these values. For amyloglucosidase, a slight increase in EAR was noted with increasing GC and CT, reaching a peak at 145 mM and 180 min, respectively. However, IT had no significant effect on AMEAR up to 9 h, after which activity recovery declined. Alcalase consistently exhibited the highest activity recovery (∼80%) across all tested conditions and remained unaffected by variations in GC, CT, and IT. This can be explained by the interaction dynamics between the enzymes and the functionalized IONPs during immobilization. At lower GC, the IONPs likely exhibited weak mechanical stability, resulting in insufficient cross-linking and a higher tendency for enzyme leaching.^21,22^ This explains the lower activity recoveries observed for cellulase and α-amylase under these conditions. As GC increased to 145 mM, the availability of aldehyde groups on the IONPs’ surface increased, facilitating stronger enzyme attachment and reducing enzyme leaching, thereby enhancing activity recovery. However, exceeding this concentration caused excessive cross-linking, likely leading to conformational changes in the enzymes that reduced their activity.^21,22^ CT followed a similar pattern, where an initial increase of up to 180 min improved enzyme–IONP interactions, leading to higher stability and activity recovery. Prolonged cross-linking times beyond 180 min appeared to distort enzyme structure or reduce enzyme flexibility, as seen in CEAR, AAEAR, and AMEAR, resulting in a decline in EAR. This suggests that overcross-linking can hinder substrate accessibility and enzyme efficiency.^9,10^ IT had varied effects among the enzymes. For CEAR and AAEAR, an IT of up to 9 h optimized adsorption and covalent bonding, maximizing activity recovery. Beyond this point, enzyme aggregation and overinteraction with the IONP surface negatively impacted activity. In contrast, AMEAR was less sensitive to IT, with declines observed only after prolonged exposure beyond 9 h, likely due to enzyme denaturation or structural changes.^23^ ALEAR demonstrated remarkable robustness, maintaining consistently high activity recovery (∼80%) across all tested conditions, reflecting its resistance to overcross-linking and suitability for immobilization applications.

**Figure 1:**
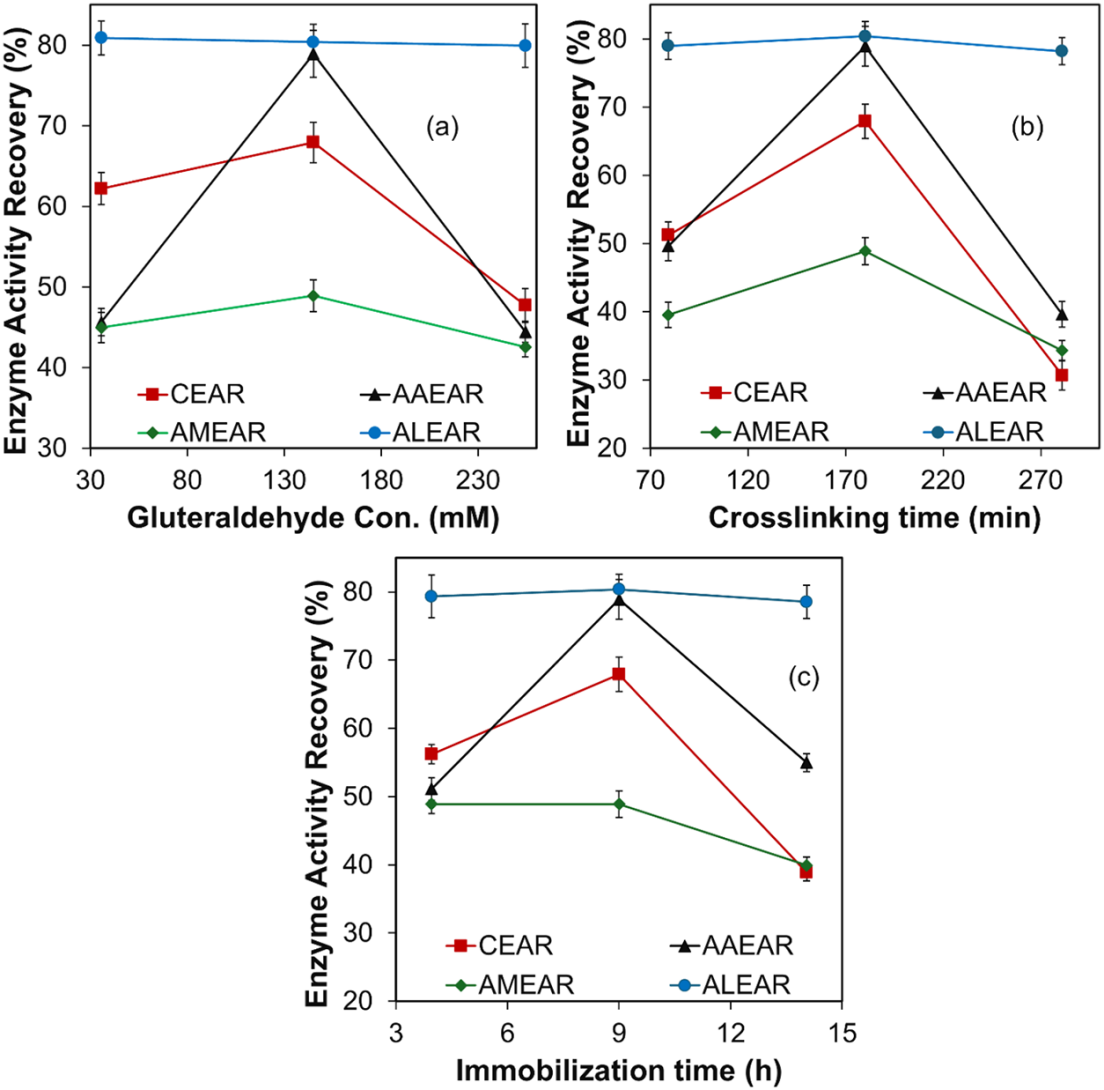
Effect of independent variables: (a) glutaraldehyde concentration, (b) cross-linking time, and (c) immobilization time on enzyme activity recovery (%). The values are the mean of two replicates (*n* = 2) with a standard deviation. CEAR, cellulase enzyme activity recovery; AAEAR, α-amylase enzyme activity recovery; AMEAR, amyloglucosidase enzyme activity recovery; ALEAR, alcalase enzyme activity recovery.

### Central Composite Design Study

To investigate the combined effects of cellulase, α-amylase, amyloglucosidase, and alcalase in the ME-MNC system, an optimization study was conducted by using a CCD. A total of 20 experimental runs were performed, during which the immobilization efficiency and EAR for each enzyme were determined, as shown in Table 2.

**Table 2:** Results of the Design of Experiments with the Actual and Coded Values for the Independent Variables^a^

		actual values			coded values				enzyme activity recovery (%)			
stages		GC (mM)	CT (min)	IT (h)	GC (mM)	CT (min)	IT (h)	immobilization efficiency (%)	cellulase	amylase	amyloglucosidase	alcalase
linear stage	**1**	**80**	**120**	**6**	**–1.00**	**–1.00**	**–1.00**	78	47	53	38	**82**
	2	210	120	6	1.00	**–**1.00	**–**1.00	79	55	58	47	79
	**3**	**80**	**240**	**6**	**–1.00**	**1.00**	**–1.00**	79	51	55	**64**	79
	4	210	240	6	1.00	1.00	**–**1.00	77	51	56	44	79
	5	80	120	12	**–**1.00	**–**1.00	1.00	78	41	55	35	80
	6	210	120	12	1.00	**–**1.00	1.00	77	58	57	52	79
	7	80	240	12	**–**1.00	1.00	1.00	78	48	50	51	79
	8	210	240	12	1.00	1.00	1.00	78	52	44	55	80
nonlinear stage	9	35.68	180	9	**–**1.68	0.00	0.00	79	62	46	45	81
	10	254.31	180	9	1.68	0.00	0.00	78	48	44	43	80
	11	145	79.09	9	0.00	**–**1.68	0.00	79	51	50	40	79
	12	145	280.91	9	0.00	1.68	0.00	77	31	40	34	78
	13	145	180	3.95	0.00	0.00	**–**1.68	79	56	51	49	79
	14	145	180	14.05	0.00	0.00	1.68	79	39	55	40	79
central stage	15	145	180	9	0.00	0.00	0.00	76	66	77	46	79
	16	145	180	9	0.00	0.00	0.00	78	65	77	48	79
	17	145	180	9	0.00	0.00	0.00	78	68	78	47	79
	18	145	180	9	0.00	0.00	0.00	79	66	79	47	80
	**19**	**145**	**180**	**9**	**0.00**	**0.00**	**0.00**	**79**	**68**	**79**	49	80
	20	145	180	9	0.00	0.00	0.00	78	67	77	48	80

a GC, glutaraldehyde concentration; CT, cross-linking time; IT, immobilization time. Bold numbers indicate the conditions at which the maximum enzyme activity recovery for each particular enzyme is obtained.

The optimization employed a quadratic model for cellulase and α-amylase and a linear model for amyloglucosidase and alcalase, enabling a comprehensive analysis of the variables involved. This approach facilitated the derivation of the following equation in terms of coded variables:

2 $$\mathrm{CEAR}=66.59+0.29\mathrm{GC}-2.57\mathrm{CT}-2.43\mathrm{IT}-3.36{\mathrm{GC}}^{2}-2.59\mathrm{GC}.\mathrm{CT}+1.57\mathrm{GC}.\mathrm{IT}-8.32{\mathrm{CT}}^{2}+0.28\mathrm{CT}.\mathrm{IT}-5.98{\mathrm{IT}}^{2}$$

3 $$\mathrm{AAEAR}=77.49-0.0756\mathrm{GC}-2.57\mathrm{CT}-0.59\mathrm{IT}-9.96{\mathrm{GC}}^{2}-1.54\mathrm{GC}.\mathrm{CT}-1.28\mathrm{GC}.\mathrm{IT}-10.1{\mathrm{CT}}^{2}-2.21\mathrm{CT}.\mathrm{IT}-7.11{\mathrm{IT}}^{2}$$

4 AMEAR=47.90+1.12GC+5.28CT−0.09IT−5.33GC.CT+3.88GC.IT−0.37CT.IT

5 ALEAR=79.50−0.47GC−0.47CT−0.12IT+0.51GC.CT+0.60GC.IT+0.34CT.IT

where CEAR, cellulase enzyme activity recovery; AAEAR, α-amylase enzyme activity recovery; AMEAR, amyloglucosidase enzyme activity recovery; ALEAR, alcalase enzyme activity recovery; GC, glutaraldehyde concentration; CT, cross-linking time; and IT, immobilization time.

### Statistical Analysis

The statistical analysis presented in Table 3 provides comprehensive insights into the EAR metrics for cellulase, α-amylase, amyloglucosidase, and alkalase in the ME-MNC system. The results reveal distinct patterns of behavior for each enzyme. For CEAR, the central point average (66.7%) was significantly higher than the linear point average (50.4%), indicating a nonlinear relationship. Significant curvature (−16.3) with a curvature confidence interval of 1.5 confirmed the importance of quadratic effects. The significant variables were CT^2^ and IT^2^, both of which exhibited negative effects. This suggests that CEAR is highly sensitive to changes in CT and IT, with optimal recovery likely occurring at intermediate values. Similarly, for AAEAR, the central point average (77.7%) exceeded the linear point average (53.4%), with a strong curvature (−24.4) and a curvature confidence interval of 1.4. This highlighted the influence of quadratic effects, with significant variables being GC^2^, CT^2^, and IT^2^, all showing negative effects. This outcome reflects strong nonlinear dependence, where α-amylase recovery decreases sharply at extreme values of GC, CT, or IT due to overcross-linking and structural stress. In contrast, AMEAR showed nearly identical averages at the central (47.5%) and linear (48.2%) points, reflecting a primarily linear relationship. Minimal curvature (0.7) within a curvature confidence interval of 1.3 indicated no significant nonlinearity. Significant variables included the interaction effects of GC × CT, GC × IT, and CT itself. These effects were negative, positive, and positive, respectively. This suggests that amyloglucosidase recovery is more influenced by the interplay between factors rather than by their individual quadratic effects. For ALEAR, the central and linear point averages were identical (79.5%), confirming a predominantly linear response. Negligible curvature (−0.02) within a curvature confidence interval of 0.96 supported this linear trend. The significant positive interaction effect of GC × IT suggests that alcalase recovery can be enhanced by simultaneously increasing the levels of GC and IT.

**Table 3:** Results of Curvature Tests with Significant Variables^a^

	enzyme activity recovery (%)			
parameters and types of tests	CEAR	AAEAR	AMEAR	ALEAR
Average of Central and Linear Points				
*Y*_Linear_	50.4	53.4	48.2	79.5
*Y*_Central_	66.7	77.7	47.5	79.5
Effect of Main Variables				
*I*_GC_	7.1	0.3	2.2	–0.9
*I*_CT_	–0.1	–4.6	10.6	–0.9
*I*_IT_	–1.0	–3.6	–0.2	–0.2
Significance Test				
standard deviation	1.1	1.0	0.9	0.7
significant variables	CT^2^, IT^2^	GC^2^, CT^2^, IT^2^	GCCT, CT, GCIT	GCIT
Significance of Curvature				
curvature	–16.3	–24.4	0.7	–0.02
curvature confidence interval	1.5	1.4	1.3	0.96
significance	yes	yes	no	no

a CEAR, cellulase enzyme activity recovery; AAEAR, α-amylase enzyme activity recovery; AMEAR, amyloglucosidase enzyme activity recovery; ALEAR, alcalase enzyme activity recovery; GC, glutaraldehyde concentration; CT, cross-linking time; IT, immobilization time.

The observed statistical patterns can be rationalized based on the chemistry of glutaraldehyde cross-linking and enzyme immobilization. For cellulase and α-amylase, the strong quadratic effects likely arise because moderate GC, CT, and IT provide sufficient aldehyde groups to enable stable multipoint covalent attachment while preserving the native enzyme conformation.^22^ At excessively high GC or prolonged CT/IT, overcross-linking or glutaraldehyde dimerization may occur, leading to steric hindrance or conformational restriction of active sites, which explains the observed negative quadratic effects.^9,24^ Amyloglucosidase, in contrast, is more sensitive to interaction terms rather than quadratic effects. This suggests that its recovery is influenced by the precise balance of GC with CT and IT: insufficient GC leads to weak enzyme binding and potential leaching, while excessive GC or CT may partially distort the enzyme’s flexible active site.^9^ For alcalase, the linear trend and the strong GC × IT effect imply that enzyme recovery is largely determined by simultaneous increases in aldehyde concentration and sufficient immobilization time, which together facilitate stable multipoint anchoring without excessive conformational stress.^22^

### Predicted versus Experimental Yield

The regression analysis of EAR for ME-MNC, as shown in Figure 2, revealed varying levels of model performance across different enzymes. AMEAR and AAEAR exhibited exceptional predictive capabilities, each with an *R* ^2^ value of 0.94, indicating strong correlations and minimal variability between experimental and predicted values (Figure 2b,c). These high *R* ^2^ values suggest that the models comprehensively capture the underlying mechanisms of EAR for these enzymes. In contrast, CEAR and ALEAR demonstrated moderate predictive performance, with *R* ^2^ values of 0.79 and 0.77, respectively (Figure 2a,d). Although these values are lower than those of AMEAR and AAEAR, they still represent substantial predictive accuracy, explaining over 75% of the variability in activity recovery. Further, these moderate *R* ^2^ values suggest that recovery mechanisms may be more complex and influenced by additional unmodeled factors, such as molecular structure, enzyme-nanoparticle interactions, or environmental variability. Thus, the moderate *R* ^2^ values for CEAR and ALEAR highlight opportunities for refinement; incorporating additional factors such as support surface density, pH, or ionic strength might improve the model fit.^21,25^

**Figure 2:**
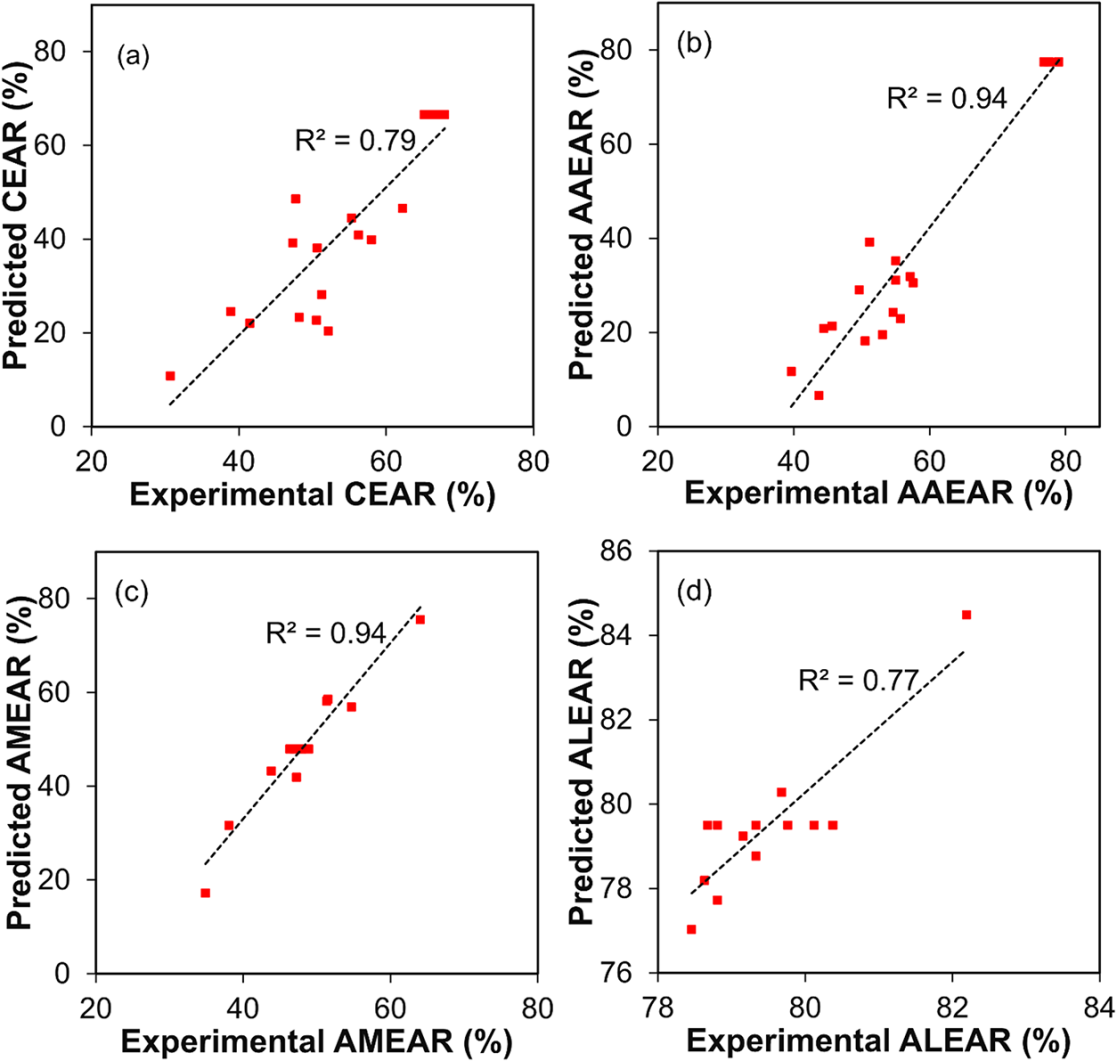
Predicted versus experimental enzyme activity recovery (%) for (a) cellulase, (b) α-amylase, (c) amyloglucosidase, and (d) alcalase. CEAR, cellulase enzyme activity recovery; AAEAR, α-amylase enzyme activity recovery; AMEAR, amyloglucosidase enzyme activity recovery; ALEAR, alcalase enzyme activity recovery.

### Analysis of Response Surface Plot

The 3D surface plots in Figures 3, 4, 5, and 6 illustrate the relationships among GC, CT, and IT on EAR for cellulase, α-amylase, amyloglucosidase, and alcalase, respectively. These plots highlight the intricate balance required to optimize enzyme immobilization. For cellulase, the maximum EAR was 68% at 145 mM GC and 180 min CT (Figure 3a and Table 2). Insufficient GC or shorter CT resulted in lower recovery due to inadequate cross-linking, while excessive GC (210 mM) or prolonged CT (240 min) reduced EAR to 52%, likely due to overcross-linking effects. IT also influenced the CEAR, with the highest recovery at 9 h. Shorter IT (6 h) resulted in incomplete enzyme attachment (47%), while longer IT (12 h) reduced recovery to 58%, likely due to partial enzyme denaturation (Figure 3b and Table 2). Cellulase recovery followed a strong nonlinear response, with maximum activity recovery at intermediate GC (145 mM) and CT (180 min), whereas both insufficient and excessive values lowered performance. This behavior can be attributed to the modular architecture of cellulase, which typically contains a catalytic domain and a carbohydrate-binding module that enhances substrate affinity.^26^ Effective immobilization requires multipoint attachment for stability, but several studies report that excessive glutaraldehyde cross-linking over-rigidify cellulase and restrict the conformational flexibility needed for substrate binding and catalysis.^26–28^ Similarly, prolonged CT has been reported to increase the density of cross-linking bridges, thereby creating steric hindrance that limits substrate diffusion into the active site—a behavior consistently observed in cellulase immobilization systems.^10,27,28^ IT also played a dual role: shorter IT led to incomplete attachment and enzyme leaching, while extended IT (12 h) increased the risk of partial enzyme denaturation due to prolonged aldehyde exposure.^9^

**Figure 3:**
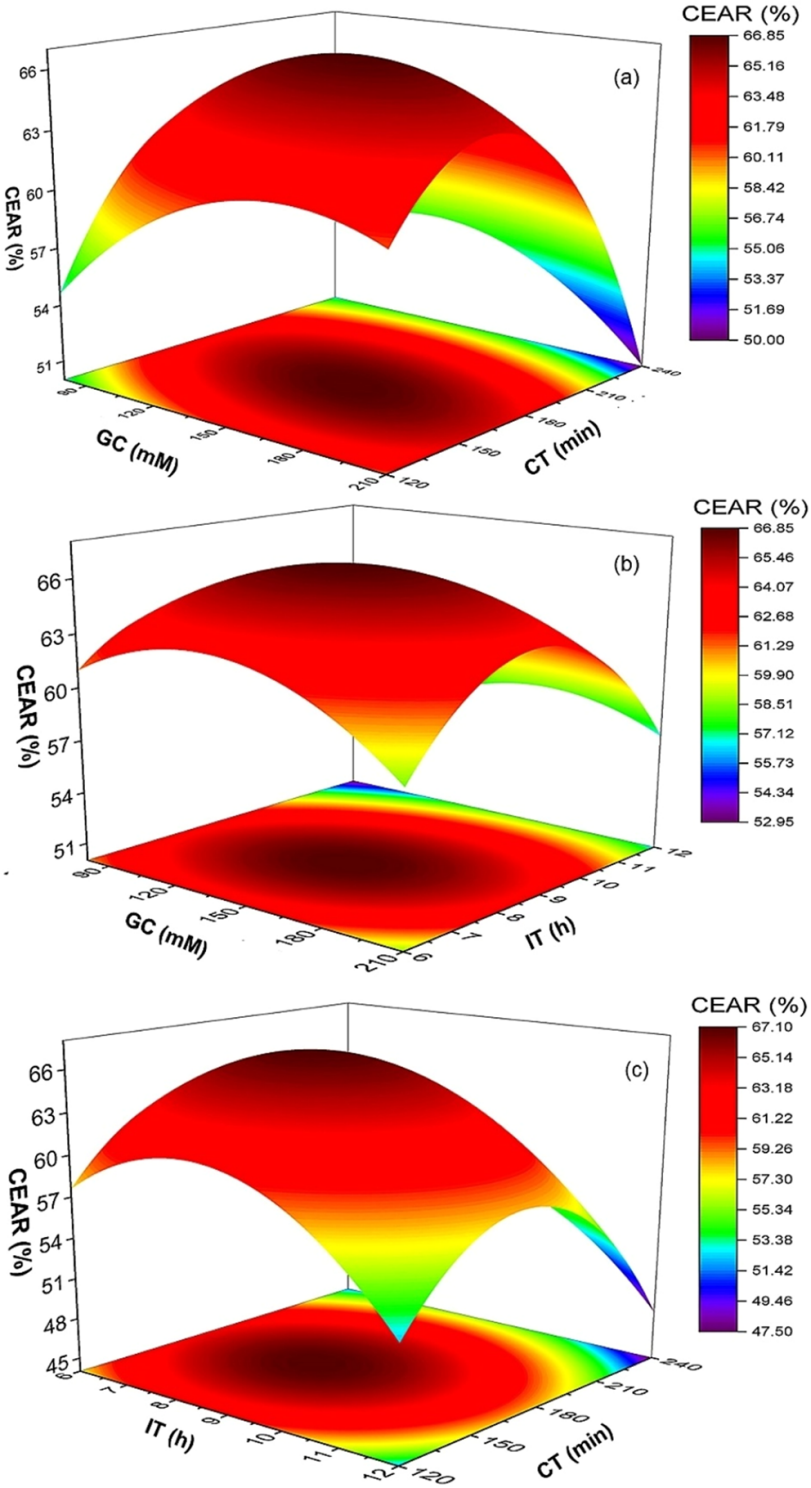
Surface plots showing the effect of glutaraldehyde concentration (GC), cross-linking time (CT), and immobilization time (IT) on activity recovery of the cellulase enzyme. CEAR, cellulase enzyme activity recovery.

**Figure 4:**
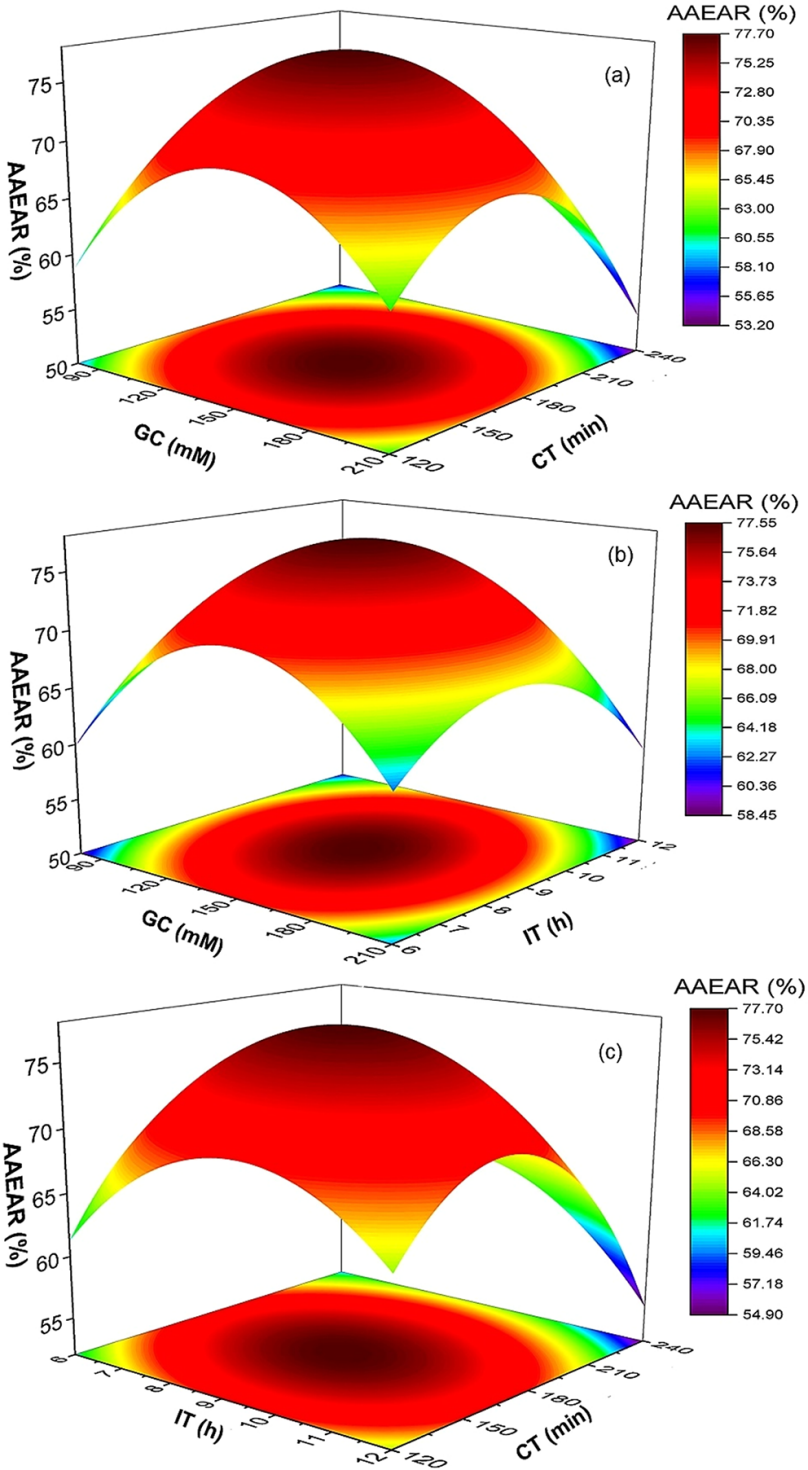
Surface plots showing the effect of glutaraldehyde concentration (GC), cross-linking time (CT), and immobilization time (IT) on activity recovery of the α-amylase enzyme. AAEAR, α-amylase enzyme activity recovery.

**Figure 5:**
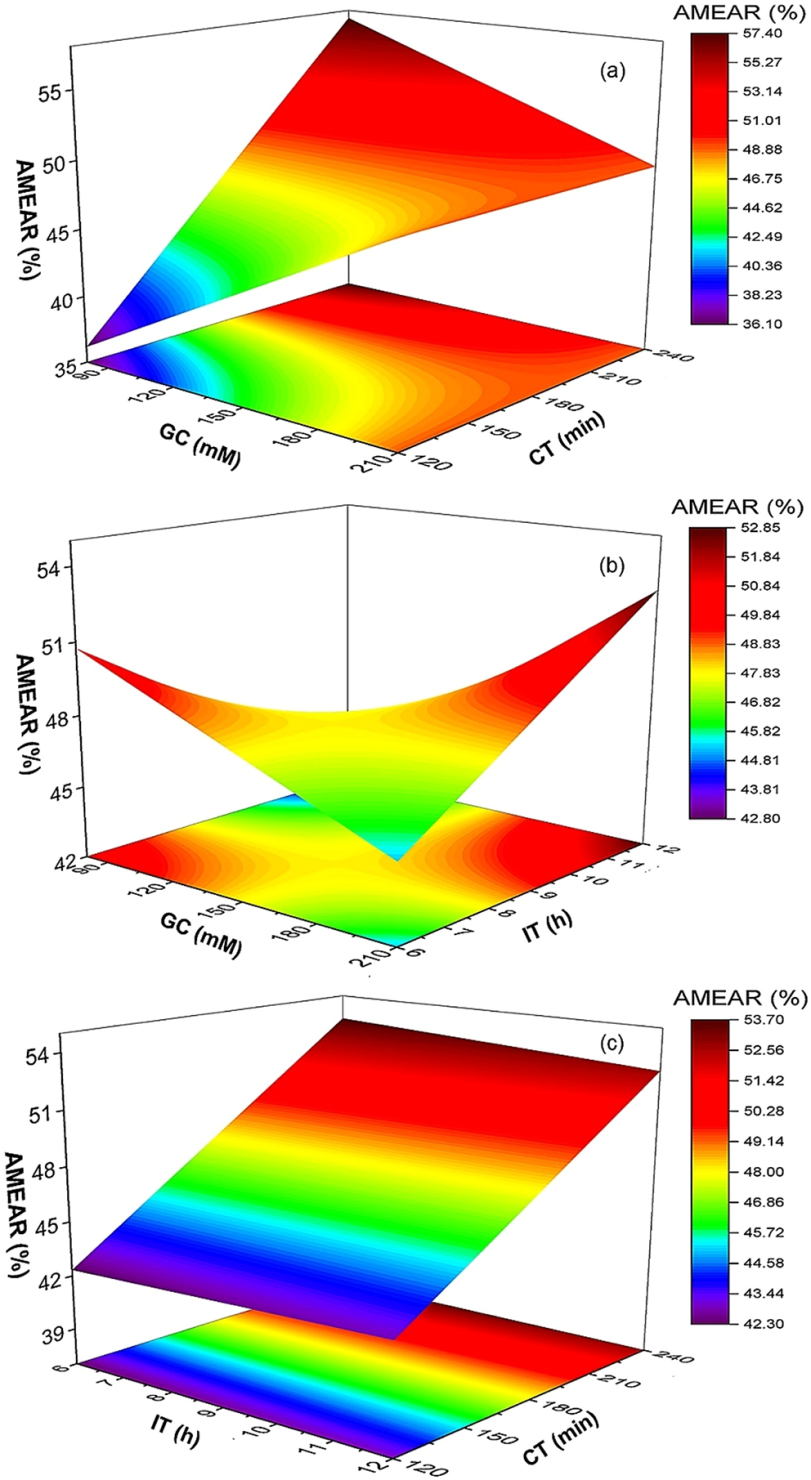
Surface plots showing the effect of glutaraldehyde concentration (GC), cross-linking time (CT), and immobilization time (IT) on activity recovery of the amyloglucosidase enzyme. AMEAR, amyloglucosidase enzyme activity recovery.

**Figure 6:**
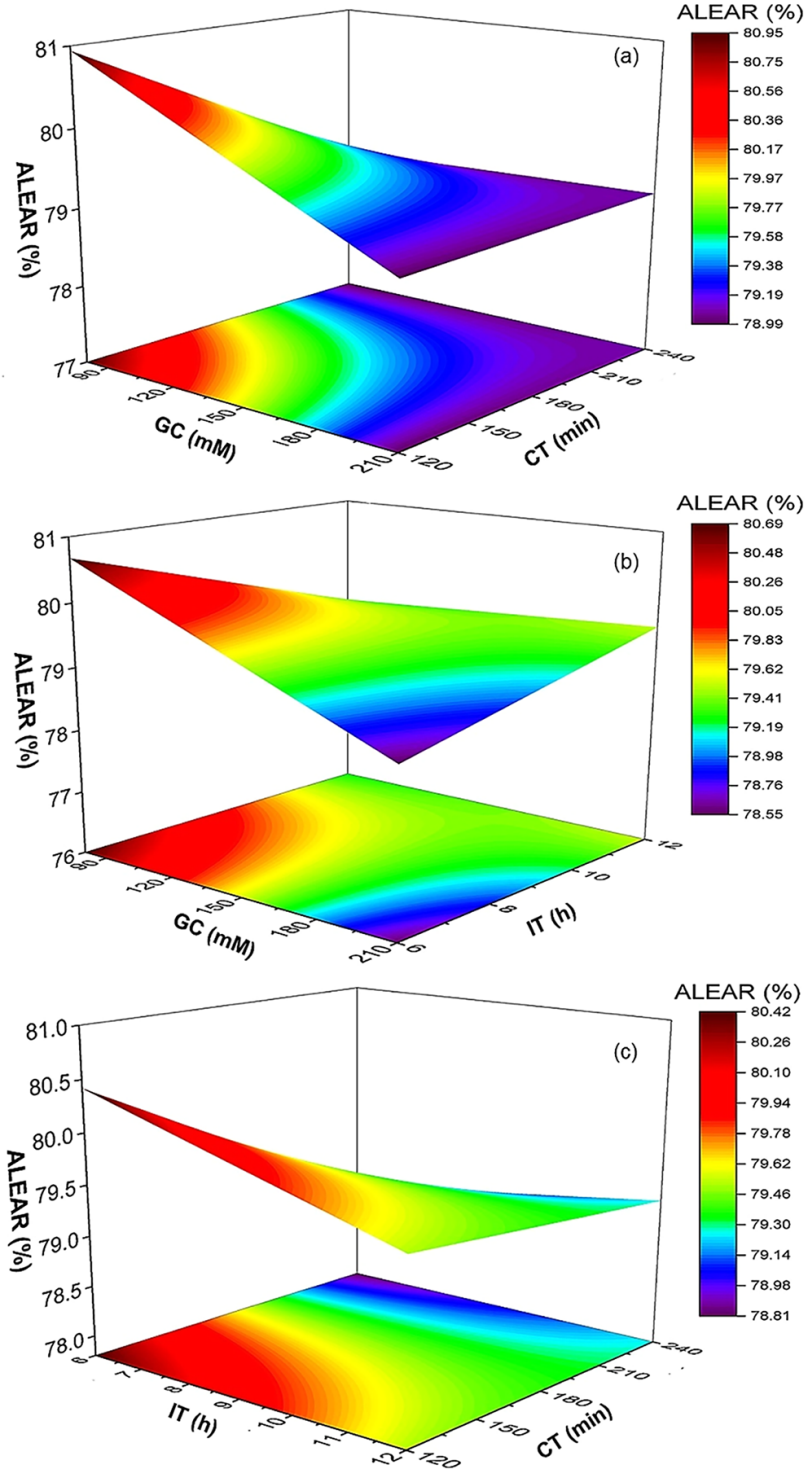
Surface plots showing the effect of glutaraldehyde concentration (GC), cross-linking time (CT), and immobilization time (IT) on activity recovery of the alcalase enzyme. ALEAR, alcalase enzyme activity recovery.

A similar trend was observed for α-amylase, which achieved a maximum EAR of 79% at 145 mM GC and 180 min CT (Figure 4a and Table 2). Shorter IT (6 h) reduced recovery to 53%, while prolonged IT (12 h) also caused a decline in the EAR (57%) (Figure 4b and Table 2). Excessive GC and CT lowered AAEAR to 44%, reflecting the negative effects of overcross-linking. α-Amylase also exhibited strong quadratic behavior, with optimal recovery (79%) at intermediate GC and CT values. Structurally, α-amylase possesses an open catalytic cleft. It relies on the flexibility of dynamic loops to mediate substrate entry, positioning, and cleavage of α-1,4 glycosidic bonds. This has been demonstrated by QM/MM molecular dynamics simulations, which revealed how loop motions and active-site rearrangements govern substrate binding, catalysis, and product release.^29^ When GC or CT concentrations were too low, cross-linking was inadequate, resulting in weak immobilization and enzyme leaching. Conversely, excessive GC and CT promoted multipoint covalent attachment near the catalytic cleft, sterically hindering substrate access and limiting the flexibility of catalytic loops, thereby impairing activity.^21,22^ IT also influenced recovery: shorter IT resulted in incomplete immobilization, whereas prolonged IT increased the risk of structural inactivation due to overexposure to glutaraldehyde. These findings are consistent with those of Salem et al.,^30^ who demonstrated that α-amylase immobilized on biomimetic magnetic nanoparticles retained high activity and reusability only when cross-linking conditions were carefully optimized. Together, these findings emphasize that α-amylase activity is highly sensitive to steric hindrance from dense cross-linking, requiring a balance between enzyme stability and catalytic accessibility.^9,31^

For amyloglucosidase, the highest EAR (64%) was obtained at 80 mM GC and 240 min of CT (Figure 5a and Table 2). Increasing GC to 210 mM reduced recovery to 44%, while extending IT to 12 h further decreased recovery to 35% (Figure 5b). Optimal recovery (64%) was achieved at 6 h of IT, but longer IT caused structural inactivation (Figure 5b and Table 2). Amyloglucosidase displayed a comparatively linear response across the tested variables, consistent with the statistical model, which indicated strong GC × CT and GC × IT interaction effects. This behavior may be attributed to the enzyme’s modular architecture: many glucoamylase/amyloglucosidase family members consist of a catalytic domain and a starch-binding domain linked by glycosylated or flexible linkers. Structural studies suggest these linkers facilitate interdomain mobility.^32^ Excessive GC likely restricted this mobility by introducing additional covalent linkages that over-rigidify the enzyme. Prolonged IT further exacerbated structural damage, which together explains the activity loss at high GC or long IT.^21^ The positive effect of longer CT at moderate GC suggests that controlled cross-linking can stabilize the enzyme against leaching or unfolding without fully constraining conformational flexibility required for catalysis.^33^ These findings underscore the importance of carefully optimizing cross-linker concentration and reaction time to balance stability with catalytic mobility.^33^ Recent work on coimmobilization strategies, including cross-linked enzyme aggregates of glucoamylase, α-amylase, and pullulanase, has demonstrated significant gains in operational stability and catalytic efficiency for glucose syrup production, further validating the importance of optimizing immobilization parameters.^31^

Unlike the other enzymes, alcalase (a subtilisin-type serine protease from *Bacillus licheniformis*) exhibited stability across conditions, with EAR values ranging from 78% to 82%. Maximum recovery occurred at 80 mM GC, 120 min CT, and 6 h of IT (Figure 6a–c and Table 2). The 3D response surfaces for alcalase show only modest sensitivity to changes in GC, CT, or IT, consistent with a predominantly linear response and minimal curvature within the tested experimental range.

This relative insensitivity can be partially attributed to structural features of subtilisin-type proteases, such as conserved calcium-binding sites, which help stabilize the protein fold and reduce susceptibility to conformational changes.^34^ Controlled multipoint covalent attachment or mild cross-linking can further rigidify the enzyme scaffold, stabilizing the tertiary structure and preserving active-site geometry, which helps explain why alcalase retained high activity across a wide range of GC, CT, and IT.^22,35^

Nevertheless, at higher GC and with prolonged CT or IT, slight declines in EAR were observed. These modest reductions likely result from increased steric crowding near the active site or subtle microconformational shifts that hinder substrate diffusion or binding. Such phenomena are well documented in CLEAs and other immobilized enzyme systems.^21,35^ The modest gains in activity at lower GC/short IT likely reflect reduced steric hindrance around the catalytic triad, whereas declines under extreme conditions are consistent with overconstraint or diffusion-limited access to exposed surface loops.^21^

The statistically significant positive GC × IT interaction suggests that simultaneous increases in both parameters enhance recovery by promoting multipoint covalent anchoring that stabilizes the enzyme without excessively blocking the active site. This interpretation aligns with prior studies on CLEAs and immobilized proteases, where careful cross-linking balances structural rigidity and catalytic accessibility.^21,35^

### Optimizing the Response

The experimental results from the optimal reactions and the predicted values of the models were compared. The optimal conditions for all enzymes were determined to be −0.63304, −0.02014, and −0.63904, corresponding to a GC of 103.82 mM, CT of 178.79 min, and IT of 7.08 h. The analysis of predicted optimal points for EAR across cellulase, α-amylase, amyloglucosidase, and alcalase revealed generally good model performance, although with some variations. Cellulase demonstrated excellent model performance, with the predicted EAR (65%) closely matching the experimental value (65%). For α-amylase, the model slightly overestimated the EAR (71% predicted vs 67% experimental), reflecting good predictive accuracy with minor discrepancies. Amyloglucosidase exhibited a slightly larger difference between the predicted (49%) and experimental (43%), indicating reasonable performance despite some deviation. Alcalase showed the highest EAR values and a close match between predicted (80%) and experimental (81%) results. The optimal point results indicate that the model effectively captures the behavior of EAR under optimal immobilization conditions, although with varying levels of accuracy across the enzymes. For CEAR, the high degree of accuracy between the predicted and experimental values aligns with the strong curvature effects observed in the statistical analysis. The model’s ability to incorporate nonlinear relationships ensures precise predictions. For AAEAR, a slight overestimation was observed. This small discrepancy indicates good model performance despite the complex nonlinear behavior and significant quadratic effects identified in the curvature analysis. The model appears to capture these complexities reasonably well. AMEAR exhibited a larger difference between the predicted and experimental values. However, its overall predictive performance remained strong. This was supported by a high *R* ^2^ value (0.94) from the regression analysis. The predominantly linear behavior noted in the curvature analysis also reinforced the model’s reliability. ALEAR showed excellent agreement between predicted and experimental EAR values, highlighting the enzyme’s predominantly linear behavior, as previously noted in the curvature analysis. The model’s straightforward linear approach aligns well with the stable recovery dynamics of alcalase. The discrepancies observed for AAEAR and AMEAR suggest opportunities for further refinement, such as incorporation of additional parameters to capture enzyme-specific structural or mechanistic factors. Despite these minor deviations, the study underscores the model’s utility as a robust tool for optimizing immobilization conditions across diverse enzymes. These insights are valuable for maximizing enzyme recovery and stability, thereby paving the way for more efficient industrial applications.

### Characterization of the Multienzyme Magnetic Nanocatalyst

Characterization of the ME-MNC was performed by using FTIR, XRD, FE-SEM, and TGA, with the results presented in Figures 7 and S1. The FTIR spectra of IONPs, amino-functionalized and glutaraldehyde-activated IONPs, and ME-MNC are presented in Figure 7a. The FTIR spectrum of amino-functionalized and glutaraldehyde-activated IONPs reveals distinct characteristic peaks that confirm successful surface modification compared with unmodified IONPs. A peak at 885.2 cm^–1^ and 1018.3 cm^–1^ corresponds to the Fe–O–Si stretching vibrations.^9,36^ The propyl group introduced by APTES is evidenced by a peak at 2935.5 cm^–1^, attributed to C–H stretching.^37^ Amino functionalization on the IONPs surface is further confirmed by a broad peak at 3377.18 cm^–1^, corresponding to N–H stretching from amino (NH_2_) groups, and peaks at 1411.8 cm^–1^ and 1558.4 cm^–1^ representing N–H bending vibrations.^9,36,38^ Glutaraldehyde grafting onto the amino-functionalized IONPs, crucial for enzyme conjugation, is confirmed by a characteristic peak at 1635.5 cm^–1^, attributed to the C=N stretching. This indicates the formation of an imine bond between the aldehyde groups of glutaraldehyde and the amino groups on the IONPs surface.^9^ In the ME-MNC spectrum, an additional peak at 1236.3 cm^–1^ appears, corresponding to C–O stretching, likely due to the enzyme binding on the IONPs. The slight shift of peaks from 1409 cm^–1^ to 1647 cm^–1^ in ME-MNC compared to activated nanoparticles suggests covalent binding of enzymes to the nanoparticle surface, highlighting successful multienzyme conjugation.^8,39^

**Figure 7:**
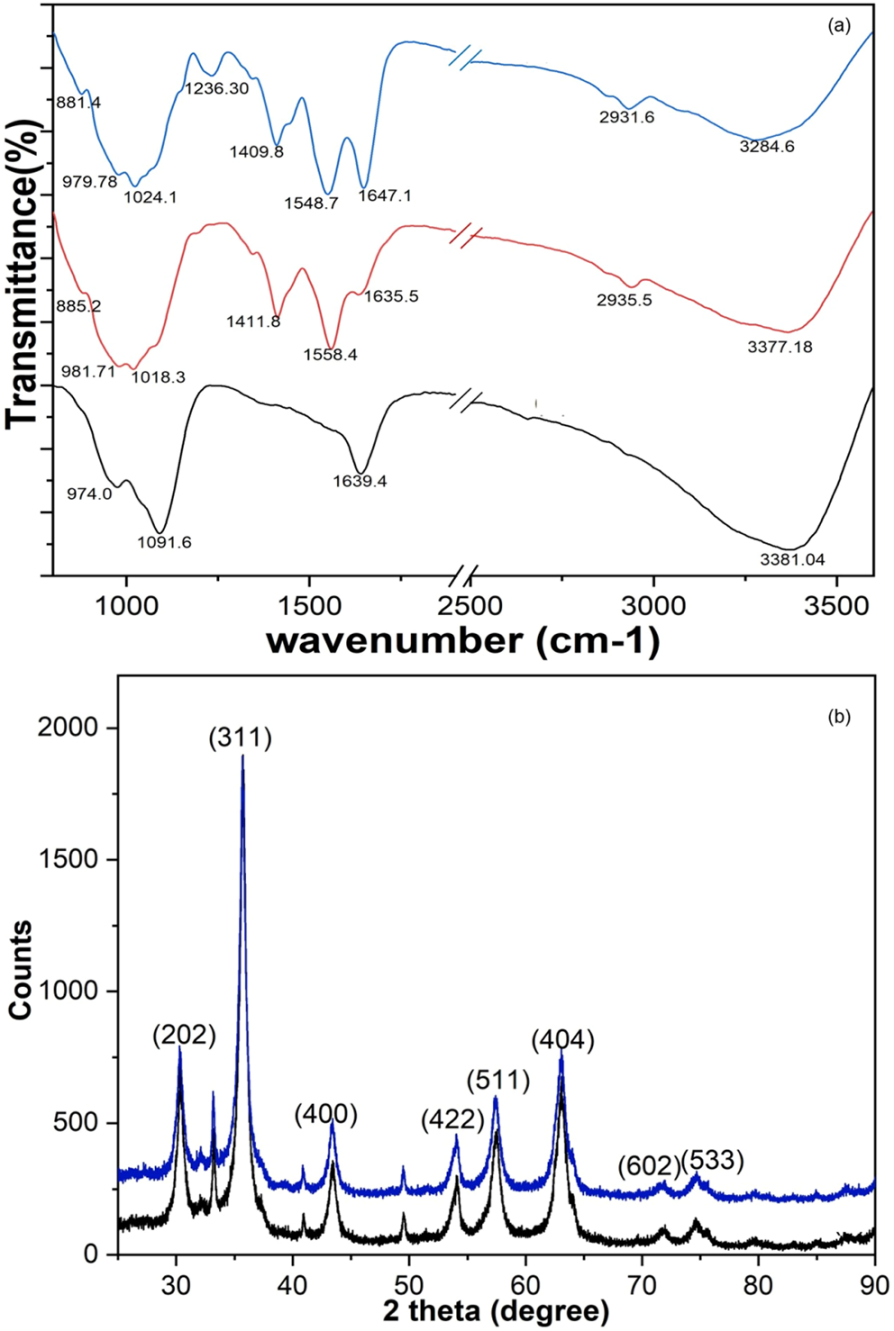
(a) FTIR spectra of the IONPs (black), amino-functionalized and glutaraldehyde-activated IONPs (red), and multienzyme magnetic nanocatalyst (blue). (b) XRD pattern of the IONPs (black) and the multienzyme magnetic nanocatalyst (blue).

The XRD analysis was conducted to determine the crystalline structure of IONPs and ME-MNC. The XRD spectra, presented in Figure 7b, reveal characteristic peaks for IONPs at 2θ values of 30.33°, 35.73°, 43.43°, 53.89°, 57.45°, 63.10°, and 74.68°, corresponding to the planes (202), (311), (400), (422), (511), (602), and (533), respectively. These peaks match standard data for Fe_3_O_4_ (COD: 9006247), confirming a cubic crystalline structure and a magnetite phase. Similarly, the ME-MNC exhibits the same characteristic peaks in its XRD spectrum, indicating that the crystalline structure of the IONPs remains unchanged during enzyme immobilization.

FE-SEM results for IONPs and ME-MNCs are presented in Figure S1a–f. The energy-dispersive spectroscopy (EDS) spectra (Figure S1a,d) indicate elemental composition. Dominant Fe and O peaks confirm that the nanoparticles primarily consist of these elements. The carbon peak in the EDS spectra is attributed to the carbon tape used for sample mounting during spectroscopic analysis. FE-SEM images (Figure S1b,e) suggest a slight increase in particle size (**∼**4 nm) for ME-MNCs compared to IONPs, potentially indicating the formation of a coating or surface layer resulting from enzyme immobilization. EDS analysis (Figure S1c,f) shows an increase in carbon content from 3.15 ± 0.8% (IONPs) to 4.76 ± 0.68% (ME-MNCs), confirming the presence of organic biomolecules from the enzyme coating. Additionally, the Fe content decreases in ME-MNCs (57.54 ± 1.65%) compared to IONPs (61.44 ± 2.69%), likely due to the enzyme layer partially masking Fe atoms. These results strongly indicate successful enzyme immobilization on the IONPs surface. A similar trend of increased carbon content and decreased Fe content after enzyme immobilization has been reported by Kaur et al.,^40^ supporting the effectiveness of this modification.

The thermal properties of IONPs and ME-MNC were analyzed by using TGA, and the weight loss curves are shown in Figure S1g. For IONPs, the total mass loss was **∼**3.4%, primarily due to the evaporation of physically and chemically adsorbed water molecules, indicating high thermal stability. In contrast, the ME-MNC exhibited a total weight loss of **∼**19.9%, occurring in distinct stages. The initial weight loss of **∼**1.9% up to 200 °C is attributed to the evaporation of physically adsorbed water.^41^ Mass loss of **∼**8.3% observed from 200 to 550 °C likely corresponds to the primary decomposition of the enzyme coatings.^41,42^ Further weight loss of **∼**8.6% from 550 to 800 °C is likely due to the combustion of residual organic coatings or stabilizing agents.^42^ Beyond 800 °C, minimal weight loss (**∼**1.1%) occurs, indicating that most organic material has decomposed, leaving an inorganic residue. These results confirm that ME-MNC contains a significant fraction of organic material, contributing to progressive weight loss, whereas IONPs remain thermally stable with minimal decomposition.

### pH and Temperature Effects on Sugar/Protein Recovery from Microalgae Using ME-MNC

The study investigated the effectiveness of ME-MNC in hydrolyzing wastewater-grown microalgae consortia biomass. The effects of pH and temperature on the release of reducing sugars and protein content were evaluated for both the ME-MNC and the FEM, as shown in Figure 8. Both systems were tested across a pH range of 4 to 10 to determine the optimal hydrolysis conditions (Figure 8a,b). The FEM achieved its highest sugar recovery (69.5 ± 2.1%) at pH 5. As pH increased from 6 to 8, sugar recovery decreased moderately (by 4.6 ± 0.5% to 27.3 ± 0.6%), while a sharp decline was observed at pH 9–10 (by 61 ± 5.5% to 83 ± 5.1%), indicating reduced enzymatic activity under alkaline conditions (Figure 8a). In contrast, the ME-MNC demonstrated a broader pH tolerance and the highest sugar recovery of 84.7 ± 1.6% at pH 6. As the pH increased from 4 to 6, sugar recovery increased by 27%, and between pH 6 and 8, sugar recovery remained stable. However, beyond pH 8, sugar release declined by 60–83%, suggesting diminished enzyme activity under more alkaline conditions (Figure 8a). Protein recovery increased progressively with pH for both systems, peaking at 25.8 ± 1.1% and 27.4 ± 0.7% at pH 10 for FEM and ME-MNC, respectively (Figure 8b). This pattern likely reflects the stability of alcalase, a protease component, under alkaline conditions, facilitating higher protein release at elevated pH levels.

**Figure 8:**
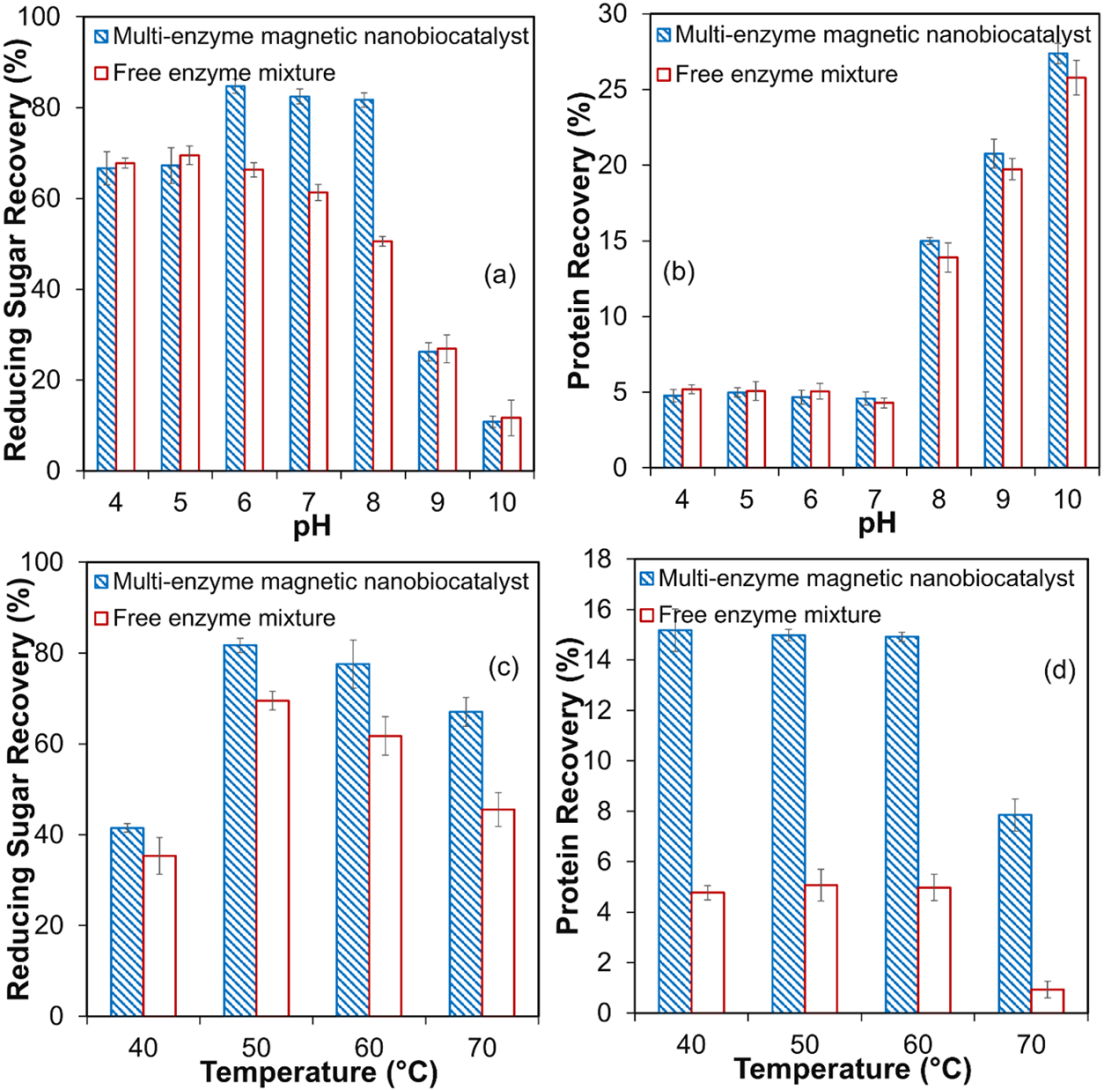
Yield of reducing sugar at different pH levels (a) and temperatures (c), and yield of protein at different pH levels (b) and temperatures (d) using the multienzyme magnetic nanocatalyst. The values are the mean of two replicates (*n* = 2) with standard deviation.

For the ME-MNC, the highest protein recovery was observed at pH 10, although sugar recovery was significantly lower at this pH. Conversely, pH 6 yielded the highest sugar recovery (84.7 ± 1.6%) but lower protein recovery (4.7 ± 0.5%). At pH 8, sugar recovery (81.7 ± 1.6%) remained close to the maximum, accompanied by a considerable increase in protein recovery (15.0 ± 0.2%). Therefore, pH 8 was selected as the optimal condition for subsequent temperature optimization, as it balanced a high sugar yield with enhanced protein release. For the FEM, based on its higher sugar recovery at pH 5, this pH was selected as optimal for temperature optimization studies.

The FEM and ME-MNC were then tested across a temperature range of 40–70 °C to determine the optimal hydrolysis conditions (Figure 8c,d). The optimal temperature for both systems was 50 °C, yielding sugar recoveries of 81.7 ± 1.6% for the ME-MNC and 69.5 ± 2.1% for the FEM. Between 40 and 50 °C, sugar recovery increased by 97% for both systems, likely due to temperature-driven enhancement of enzymatic activity, which facilitates carbohydrate hydrolysis in the microalgae biomass. Beyond 50 °C, sugar recovery remained stable at 60 °C for the ME-MNC but decreased by 11% in the FEM, highlighting the superior thermal stability of the immobilized enzymes. At 70 °C, sugar recovery declined significantly in both systems, with reductions of 17% for the ME-MNC and 34.5% for the FEM, indicating the onset of enzyme thermal denaturation, which reduces the catalytic efficiency at elevated temperatures (Figure 8c). Protein recovery showed minimal variation from 40 to 60 °C in both systems, suggesting that alcalase retained its activity across this range. However, at 70 °C, protein recovery dropped significantly—by 48% for ME-MNC and 80% for the FEM—indicating thermal denaturation of the protease enzymes and reduced ability to hydrolyze proteins effectively at this elevated temperature (Figure 8d).

The enhanced pH tolerance of ME-MNC can be attributed to the covalent immobilization of enzymes on the IONPs. Covalent bonding likely stabilizes the enzyme structure and minimizes conformational changes that would otherwise reduce activity in free enzymes, particularly under less favorable pH conditions.^9,43^ The use of glutaraldehyde as a cross-linking agent may also modify the enzyme’s microenvironment, potentially influencing its pH optimum.^9^ In some cases, immobilization has been observed to shift the optimal pH for the catalytic activity. For example, α amylase, glucoamylase, and pullulanase coimmobilized on IONPs exhibited a broadened pH-activity profile.^9^ Similarly, cellulase and lysozyme immobilized on glutaraldehyde-activated IONPs demonstrated improved stability across different pH values.^10^ This shift, from pH 5 in FEM to pH 6–8 in the ME-MNC, appears to enhance enzyme stability and function at higher pH values.^9^ Additionally, covalent immobilization may improve enzyme orientation on the IONPs, facilitating substrate access to active sites and reducing steric hindrance.^9,43^ The ME-MNC demonstrated superior performance across all pH and temperature conditions, with higher yields of both reducing sugars and protein compared with the FEM (Figure 8). This suggests that coimmobilization on IONPs not only enhances enzyme stability and activity but also likely improves resistance to environmental variations through favorable orientation and reduced diffusion limitations. The current study also compared its results to those reported in the literature, as shown in Table 4. It achieved an 81.7% sugar recovery, comparable to the 91% reported by Velmurugan and Incharoensakdi^8^ and similar to the 83% obtained using polyurethane foam by Rempel et al.^44^ Unlike other studies that primarily focused on sugar, this work uniquely reports a protein recovery of 15%. Furthermore, the enzyme diversity in this study enables broader substrate hydrolysis, underscoring its novel approach to microalgae biomass processing.

**Table 4:** Comparison of This Study with the Literature Study

microalgae sp.	enzymes	immobilization material	sugar recovery (%)	protein recovery (%)	references
*Chlorella* sp.	cellulase	polyacrylonitrile nanofibrous membranes	63.2		^11^
*Spirulina platensis*	α-amylase and amyloglucosidase	polyurethane foam	83		^44^
*Chlorella* sp. CYB2	cellulase enzyme complex	MgO-Fe_3_O_4_	91		^8^
*Scenedesmus* and *Tribonema* sp. consortia	cellulase, amylase, amyloglucosidase, and alcalse	iron oxide nanoparticles	81.7	15	current study

### Reusability of the Immobilized Enzyme System

The reusability of the immobilized enzyme system (ME-MNC) was evaluated over five consecutive hydrolysis cycles to assess its catalytic stability and efficiency retention, as shown in Figure 9. The reducing sugar recovery displayed a gradual decline with each cycle. Between cycles 1 and 2, an 8.3% reduction in sugar release was observed. By cycle 5, the cumulative reduction in the reducing sugar yield reached 18.9% compared to the first cycle. In contrast, protein release remained relatively stable, showing only a 4.4% decrease by cycle 5, with consistent stability up to cycle 3.

**Figure 9:**
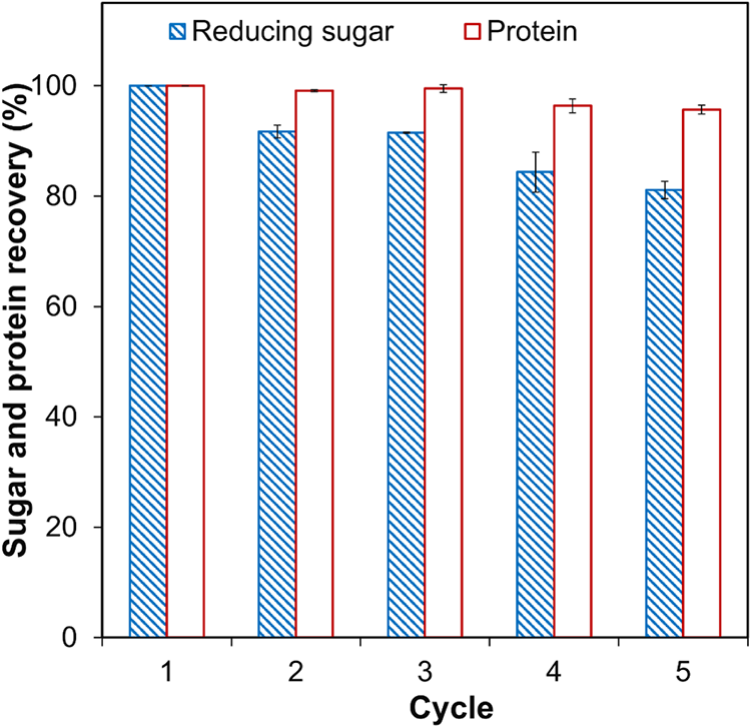
Reusability study of ME-MNC: sugar and protein recovery over five cycles. The values are the mean of two replicates (*n* = 2) with standard deviation.

The initial 8.3% reduction in sugar release, as shown in Figure 9, suggests a minor loss in catalytic activity, likely due to slight enzyme leaching or structural changes during handling and washing.^10,45^ The moderate decline in hydrolytic efficiency over five cycles indicates gradual enzyme inactivation or detachment from nanoparticles, though this could potentially be minimized by optimizing immobilization techniques or adjusting the operational conditions to enhance stability. The relatively stable protein recovery suggests that alcalase maintained its structural integrity and catalytic function despite repeated washing and reuse. These findings highlight the favorable reusability of the immobilized enzyme system, retaining 81.1 ± 1.6% sugar recovery efficiency and 95.7 ± 0.8% protein recovery efficiency after five cycles. This durability underscores its potential for repeated biomass hydrolysis applications, offering a promising reduction in enzyme consumption and operational costs for long-term industrial use.

## Conclusions

The successful development and optimization of the multienzyme magnetic nanocatalyst (ME-MNC) in this study offer a promising solution for the efficient hydrolysis of microalgae consortia cultivated in dairy wastewater. By immobilization of cellulase, α-amylase, amyloglucosidase, and alcalase onto amino-functionalized iron oxide nanoparticles, the ME-MNC significantly enhanced enzyme stability and reusability. Optimization using the Central Composite Design ensured high activity recovery with experimental results closely matching predicted values. The ME-MNC achieved superior sugar recovery (81.7 ± 1.6%) compared to that of the free enzyme mixture (69.5 ± 2.1%). The catalyst exhibited excellent stability across a broad pH and temperature range and retained high efficiency after multiple reuse cycles, demonstrating its potential for sustainable biomass conversion. These findings highlight the ME-MNC as a promising biocatalyst for enhancing microalgae hydrolysis, offering a cost-effective and environmentally friendly approach for bioresource utilization.

## Supplementary Material



## Notes

Preparation of the multienzyme magnetic nanocatalyst (ME-MNC); comprehensive characterization details; FE-SEM-EDS images; and thermogravimetric analysis (TGA) curves of IONPs and ME-MNC. (PDF)
