# Supplementary material for: A Multienzyme Magnetic Nanocatalyst for Efficient, Sustainable Hydrolysis of Wastewater-Grown Microalgae Consortia
Source: ACS Sustain Chem Eng. 2025 Dec 17;14(1):123–35. doi: 10.1021/acssuschemeng.5c08509 (PMC12801979; doi:10.1021/acssuschemeng.5c08509)
Supplement: Supplementary file 1 [file sc5c08509_si_001.pdf]

Supporting Information for Publication

A multi-enzyme magnetic nanocatalyst for efficient, sustainable hydrolysis of wastewater-grown microalgae consortia

*Suvidha Gupta, Jorge M. Marchetti**

Faculty of Science and Technology, Realtek, Norwegian University of Life Sciences, Drøbakveien 31, 1432 Ås, Norway

*Corresponding Author: E-mail: Jorge.mario.marchetti@nmbu.no

Total number of pages (including cover page): 4

Total number of Figures: 1

MATERIALS AND METHODS

Preparation of the multi-enzyme magnetic nanocatalyst (ME-MNC)

The multi-enzyme magnetic nanocatalyst (ME-MNC) was synthesized by co-immobilizing cellulase, α-amylase, amyloglucosidase, and alcalase onto amino-functionalized iron oxide nanoparticles (IONPs) activated with glutaraldehyde. Briefly, 1 g of IONPs was amino-functionalized at 70 ˚C for 5 h, followed by glutaraldehyde activation as described by Talekar et al.[9] The activated nanoparticles were magnetically separated and washed repeatedly with deionized water to remove excess glutaraldehyde, followed by washing with 50 mM acetate buffer containing 10 mM $CaCl_2$ (pH 5). For enzyme co-immobilization, equal volumes (0.5 mL) of cellulase, α-amylase, amyloglucosidase, and alcalase solutions (adjusted to the same protein concentration) were combined to obtain a 2 mL enzyme mixture, which was incubated with 10 mg of glutaraldehyde-activated IONPs at 25 ˚C under stirring. After immobilization, the ME-MNC was magnetically separated, washed with acetate buffer to remove unbound enzymes, freeze-dried, and stored at 4 ˚C until further use. Preparation parameters, including glutaraldehyde concentration (GC), cross-linking time (CT), and immobilization time (IT), were optimized based on enzyme activity recovery (EAR). To determine immobilization efficiency, the supernatants from the washing steps were analyzed for protein content using the Bradford assay.[13] The absence of detectable protein indicated complete immobilization, and immobilization efficiency (%) was calculated using Equation (1).

$$\text{Immobilization efficiency} (\%) = \frac{\text{Total protein added} - \text{Protein in supernatant}}{\text{Total protein added}} \times 100 \quad (1)$$

Characterization of the multi-enzyme magnetic nanocatalyst. FTIR analysis was carried out for IONPs, amino-functionalized and glutaraldehyde-activated IONPs, and ME-MNC to determine the functional groups. ATR-FTIR analysis was conducted using a Vertex 70 FTIR spectrophotometer equipped with a diamond ATR crystal accessory. Spectra were collected at a resolution of 4 cm^{-1}, accumulating 32 scans over a wavenumber range of 600 to 4000 cm^{-1}. A DTGS (Deuterated Triglycine Sulfate) detector was used for signal detection. Background spectra were collected and automatically subtracted from each sample spectrum. Samples were placed directly on the ATR crystal without additional preparation, ensuring good contact between the sample and the crystal surface. The data were processed using Orange software. XRD analysis was conducted at the XRD facility of the MINA department, NMBU, using a Bruker D8 Advance diffractometer with Cu Kα radiation at a generator voltage of 40 kV and 40 mA. Diffraction data for the IONPs and ME-MNC were collected over a 2θ range of 20° to 90° with a step size of 0.01° in Bragg-Brentano reflection mode. Phase identification and crystallite size estimation were performed using Bruker Diffrac.Eva software, with diffraction patterns matched against the Crystallography Open Database (COD) reference database. The morphology of IONPs and ME-MNC was determined using a Zeiss Gemini 450 SEM. Elemental composition was determined by EDS using a Zeiss Supra 55 VP SEM. Samples were prepared by mounting nanoparticles on

aluminum stubs with carbon tape. TGA of IONPs and ME-MNC was performed using a TGA/DSC 3+ analyzer (Mettler Toledo).

RESULTS AND DISCUSSION

Characterization of the multi-enzyme magnetic nanocatalyst

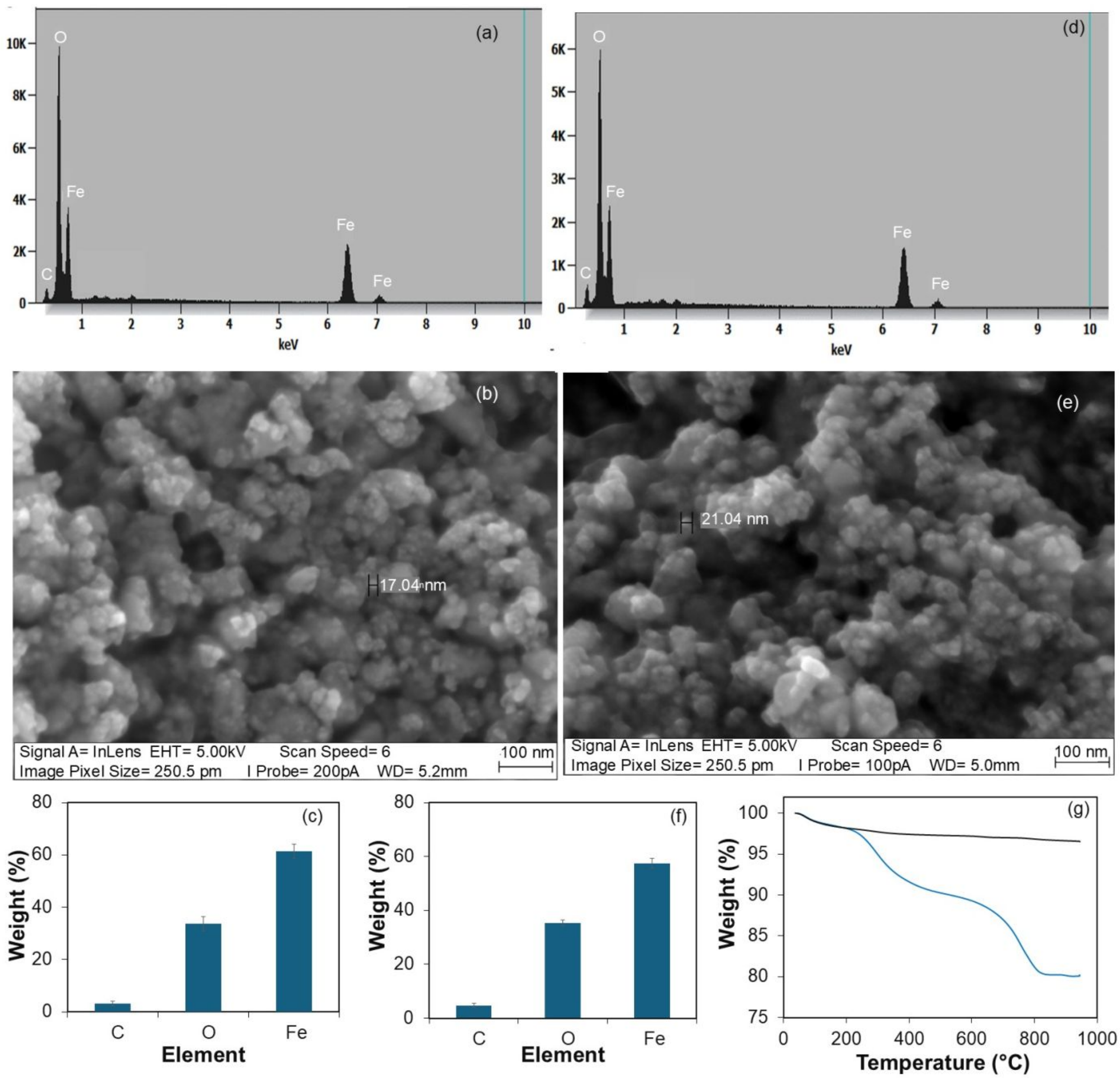

Figure S1. FESEM-EDS analysis of IONPs (a- EDS spectra, b- microscopic image, c- weight percentage) and ME-MNC (d- EDS spectra, e- microscopic image, f- weight percentage), g TGA curves of IONPs (black) and ME-MNC (blue).
